# Overview of Cancer Control in Armenia and Policy Implications

**DOI:** 10.3389/fonc.2021.782581

**Published:** 2022-01-11

**Authors:** Karen Bedirian, Tigran Aghabekyan, Arianna Mesrobian, Shant Shekherdimian, Davit Zohrabyan, Liana Safaryan, Lilit Sargsyan, Armen Avagyan, Lilit Harutyunyan, Astghik Voskanyan, Artashes Tadevosyan, Davit Melik-Nubaryan, Parandzem Khachatryan, Tatul Saghatelyan, Mher Kostanyan, Hovhannes Vardevanyan, Marine Hovhannisyan, Tamara Sarkisian, Karine Sargsyan, Davit Babikyan, Armen Tananyan, Samvel Danielyan, Armen Muradyan, Gevorg Tamamyan, Samvel Bardakhchyan

**Affiliations:** ^1^ Pediatric Cancer and Blood Disorders Center of Armenia, Hematology Center After Prof. R. Yeolyan, Yerevan, Armenia; ^2^ Faculty of Medicine, Yerevan State Medical University, Yerevan, Armenia; ^3^ Department of Pediatric Surgery, Yerevan State Medical University, Yerevan, Armenia; ^4^ Department of Public Health, Yerevan State Medical University, Yerevan, Armenia; ^5^ Ministry of Health of the Republic of Armenia, Yerevan, Armenia; ^6^ Department of Pediatric Surgery, University of California Los Angeles, Los Angeles, CA, United States; ^7^ Department of Oncology, Yerevan State Medical University, Yerevan, Armenia; ^8^ Clinic of Adults’ Solid Tumors, Hematology Center After Prof. R. Yeolyan, Yerevan, Armenia; ^9^ Department of Pediatric Oncology and Hematology, Yerevan State Medical University, Yerevan, Armenia; ^10^ Armenian Pediatric Hematology and Oncology Group, Yerevan, Armenia; ^11^ Clinic of Chemotherapy, Mikayelyan Institute of Surgery, Yerevan State Medical University, Yerevan, Armenia; ^12^ Armenian Association of Hematology and Oncology, Yerevan, Armenia; ^13^ Young Oncologists Group of Armenia, Yerevan, Armenia; ^14^ Clinic of Adults’ Hematology, Hematology Center After Prof. R. Yeolyan, Yerevan, Armenia; ^15^ Department of Public Health and Healthcare Organization, Yerevan State Medical University, Yerevan, Armenia; ^16^ Division of Clinical Affairs, Yerevan State Medical University, Yerevan, Armenia; ^17^ Department of Clinical Pathology, Yerevan State Medical University, Yerevan, Armenia; ^18^ “Histogen” Armenian-German Scientific Center of Pathology, Yerevan, Armenia; ^19^ Department of Radiation Oncology, National Center of Oncology, Yerevan, Armenia; ^20^ Department of Oncology, National Center of Oncology, Yerevan, Armenia; ^21^ Department of Radiology, Armenian-American Wellness Center, Yerevan, Armenia; ^22^ Faculty of Public Health, Yerevan State Medical University, Yerevan, Armenia; ^23^ Department of Medical Genetics, Yerevan State Medical University, Yerevan, Armenia; ^24^ Center of Medical Genetics and Primary Health Care, Yerevan, Armenia; ^25^ International Biobanking and Education, Medical University of Graz, Graz, Austria; ^26^ National Medical Research Radiological Centre of the Ministry of Health of the Russian Federation, Moscow, Russia; ^27^ Laboratory of Molecular Genetic, Center of Medical Genetics and Primary Health Care, Yerevan, Armenia; ^28^ Hematology Center after Prof. R. Yeolyan, Yerevan, Armenia; ^29^ Department of Urology, Yerevan State Medical University, Yerevan, Armenia; ^30^ Institute of Cancer and Crisis, Yerevan, Armenia

**Keywords:** cancer policy, cancer epidemiology, cancer risk factors, cancer prevention, early detection of cancer, cancer treatment, developing countries, Armenia

## Abstract

Cancer is the second leading cause of death in Armenia. Over the past two decades, the country has seen a significant rise in cancer morbidity and mortality. This review aims to provide up-to-date info about the state of cancer control in Armenia and identify priority areas of research. The paper analyzes published literature and local and international statistical reports on Armenia and similar countries to put numbers into context. While cancer detection, diagnosis, and treatment are improving, the prevalence of risk factors is still quite high and smoking is widespread. Early detection rates are low and several important screening programs are absent. Diagnosis and treatment methods are not standardized; there is a lack of treatment accessibility due to insufficient government coverage and limited availability of essential medicines. Overall, there is room for improvement in this sector, as research is limited and multidisciplinary approaches to the topic are rare.

## Introduction

With an overall rise in the incidence of noncommunicable diseases in the world, cancer has become one of the leading causes of morbidity and mortality (1). A significant part of the burden of this disease is shared by low and middle-income countries (LMICs). Some projections estimate that by 2030, nearly 70% of all cancer cases will be diagnosed in these countries (1).

In recent years, there has been a sharp increase in cancer-related epidemiologic indicators in Armenia. Cancer is now the second leading cause of death in Armenia, accounting for 21% of all deaths (2, 3). The incidence and prevalence of cancer have increased significantly over the past decade, with an even greater increase expected by 2040 (2, 4).

Armenia also compares poorly with the countries of the region and globally (2). It has been among the top five countries with the highest incidence and mortality of cancer in the region of Western Asia (2). Taking into account the country’s limited resources in the treatment and management of cancer, it is essential that Armenia strengthens its prevention and early detection strategies which would prove more efficient in resource management (5). This is especially important when considering the lifestyle and habits of the people in Armenia and the region, which are, in general, far from healthy (6).

This review is meant to provide a comprehensive description of the situation in Armenia regarding different aspects of cancer, such as incidence, prevalence, morbidity, mortality, prevention, early detection, and treatment. We aim to help identify priority areas of research and improvement in this field.

## Materials and Methods

The review is based on published literature and national and international reports. In addition, experts were consulted to confirm the information provided in older reports and to identify areas where research is lacking. Epidemiology data were graphed and their trends analyzed using the Joinpoint Regression Program version 4.9.0.0 which utilizes the permutation test to choose the best Jointpoint model that fits the data (7). It is worth mentioning that data from the National Institute of Health (NIH) are not based on a population-based cancer registry, as Armenia does not have one. GLOBOCAN data are estimated based on the cancer registries of neighboring countries (2).

## Results and Discussion

### Overview of the Health System

Armenia is a middle-income country located between Europe and Asia, with a population of 2.9 million people (8). It has a GDP per capita of $4,600, which is slightly above the lower threshold of upper-middle-income countries (9). Armenia acquired independence following the dissolution of the Soviet Union in 1991 (10). It suffered a devastating earthquake in 1988, followed by a years-long war, during which health care reforms were not considered a priority (10, 11). After gaining independence, Armenia’s health care system underwent a steep transformation from a centralized Soviet system, in which all levels of health care were publicly financed, to a highly fragmented one, financed mainly out-of-pocket (10). Government expenditure on health constitutes about 1.24% of the GDP, compared to an average of 4% for upper-middle-income countries (12, 13). As a result of this and the lack of significant voluntary health insurance coverage, out-of-pocket health expenditure accounts for about 84% of the current health expenditure (12). This is well above the World Health Organization’s (WHO) recommended maximum of 20% (14). and it surpasses the out-of-pocket health expenditure rates of all other countries worldwide (2017 estimates) (12).

### Organization of Cancer Care

#### Financing

Total expenditure on cancer constitutes around 1.9% of Armenia’s current health expenditure (5). European countries spend considerably more on cancer, with a corresponding average of 6% (15). Several state laws and initiatives in Armenia do, however, aim to cover the cost of cancer care (16).

Surgical treatment and radiation therapy are provided free of charge for all cancer patients through a program launched by the Government in 2019 (17). However, the allocated budget for this program did not correspond to the demands of the public sector which has led to a rise in treatment wait times (18). Furthermore, the inadequate budget may have additional downstream consequences such as the inability to assure high-quality care, along with the potential outmigration of clinicians from the public sector (19).

While outpatient and inpatient cancer care are fully covered by the government for all cancer patients, only vulnerable groups receive full coverage for chemotherapeutic treatment (16). However, ‘full coverage’ is not actually achieved because there is a spending limit on chemotherapy even for vulnerable groups (16). The government covers around $750-worth of chemotherapy per year for vulnerable individuals and only half of that for non-vulnerable persons.

#### Infrastructure

Overall, 14 centers provide chemotherapy and three centers provide radiation therapy in Armenia. Many medical centers provide surgical interventions for cancer patients, but only eight of them provide specialized oncological surgery. Pediatric oncology services are provided in only one center in the country. Six of the fourteen available centers that provide cancer care are publicly owned (20, 21). Armenia does not have the issue of overloaded facilities; difficulties arise primarily due to the ill distribution of these facilities. The majority of cancer care facilities are situated in the capital city, Yerevan, where one-third of the population resides. Outside of Yerevan, there are only two centers that provide chemotherapy and oncological surgery (Gyumri and Vanadzor). There are no centers that provide radiotherapy outside of Yerevan. Thus, regions that are sparsely populated do not have equal access to cancer care facilities, contributing to the delay or complete neglect of necessary treatment. On the other hand, having cancer centers in these sparsely populated regions is not sustainable. Therefore, difficulties in access to care should be countered by facilitating the transport and stay of patients in Yerevan, creating outreach clinics for Yerevan-based clinicians to visit regularly, and expanding the scope of telemedicine.

#### Workforce

In Armenia, there are 89 medical oncologists, 18 hematologists, 11 radiation oncologists, 51 surgical oncologists, and 11 pediatric oncologist-hematologists (3). Thus, one adult specialist provides care for about 73-88 new malignant cases per year, and one pediatric oncologist-hematologist provides care for about 8 new cases per year (3). These numbers are similar to many Eastern European countries, such as Ukraine and Hungary, and they indicate a surplus of oncologists (22). However, the geographic distribution of this workforce is not ideal (3). While several provinces have a shortage of oncologists, two of Armenia’s eleven provinces have a complete lack of medical oncologists (3). Similarly, all pediatric oncologists and hematologists are located in the capital city Yerevan (3).

The education of specialists consists of a 6-year basic medical training followed by a 3-year residency training program in either hematology, oncology, or pediatric hematology-oncology. Graduates of the oncology residency program can officially work as medical, radiation, or surgical oncologists. However, they usually receive further training in the case of the latter two. Every year, around 8-10 students enroll in the oncology residency program. It is worth mentioning that the department of pediatric oncology and hematology of Yerevan State Medical University (YSMU) has recently been created in 2019. Armenia is among the first in former Soviet countries that created a unified “pediatric hematologist-oncologist” specialty, and currently, 10 fellows are enrolled in this program.

Several professional medical associations have been established in the past decade, these associations actively organize scientific events to ensure the professional development of specialists.

### Cancer Statistics and Epidemiologic Measures

**Figure 1:**
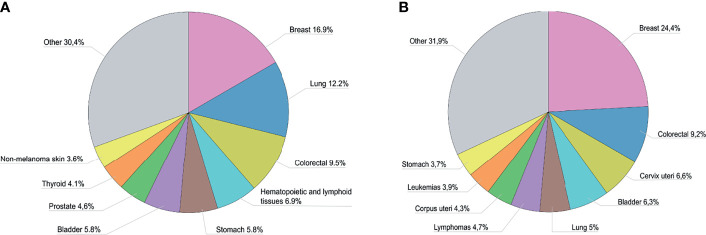
**(A)** Proportion of new cases by cancer type in Armenia in 2019^a.^ **(B)** Proportion of prevalent cases by cancer type in Armenia in 2019^a^. ^a^Data are adapted from the Statistical Yearbook of Armenia: Health and Healthcare 2020 (3).

According to the Ministry of Health of Armenia, the crude cancer incidence rate in 2019 was 266.9 per 100,000 people (3). The cancers with the highest incidence rates were breast, colorectal, and cervical, among females and lung, bladder, and colorectal among males (**Figure 1A**, **Table 1A of the supplement**). The crude rate of prevalent cancer cases was 1699.5 per 100,000 people, with a female predominance. Breast, colorectal, cervical, uterine, bladder, and lung cancers were the most prevalent (**Figure 1B**, **Table 1B of the supplement**). The crude mortality rate in 2019 was 183.4 per 100,000 people — slightly higher among men than women (3).

#### Analysis of Trends

**Figure 2:**
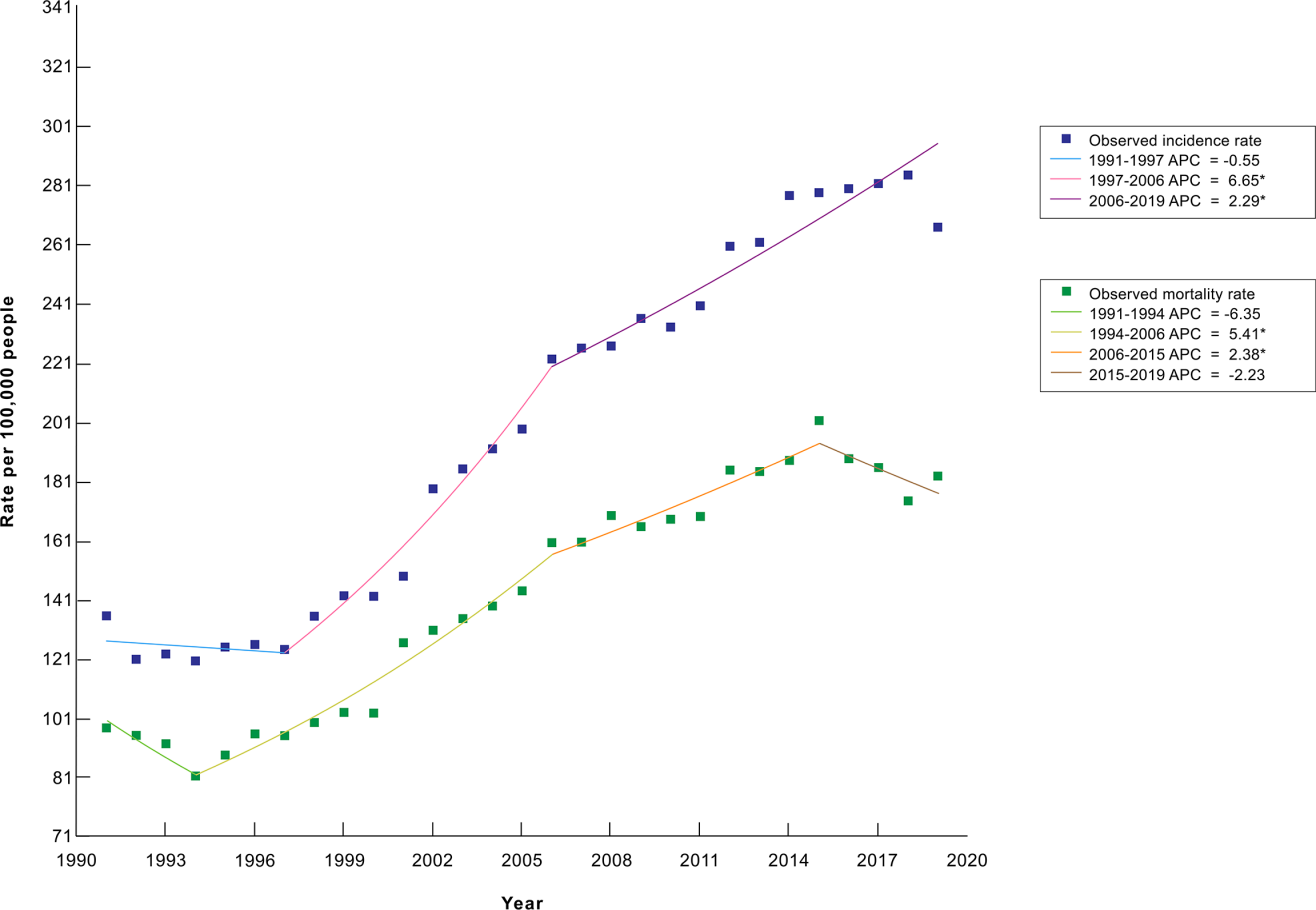
Trends in the crude cancer incidence rate and the crude cancer mortality rate from 1991 to 2019 in Armenia, per 100,000 people^a^. ^a^Data are adapted from the Statistical Yearbook of Armenia: Health and Healthcare 2020 (3) and the Statistical Yearbook of Armenia 1993-1994, 1995-1996, 2001, 2004, and 2009 (23–27). *Indicates that the Annual Percent Change (APC) is significantly different from zero at the alpha = 0.05 level.

**Figure 3:**
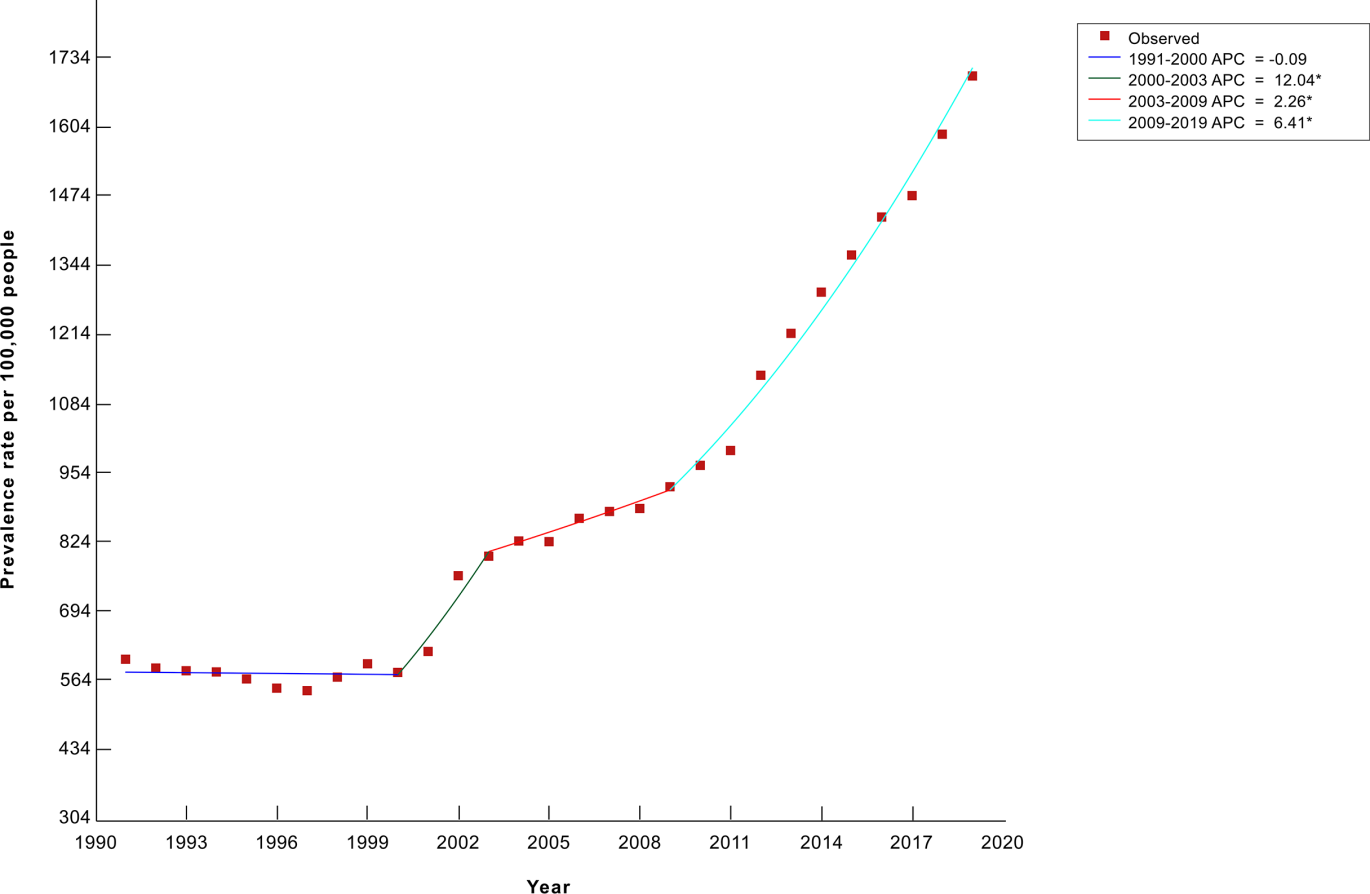
Trends in the crude cancer prevalence rate from 1991 to 2019 in Armenia, per 100,000 people^a^. ^a^Data are adapted from the Statistical Yearbook of Armenia: Health and Healthcare 2020 (3). *Indicates that the Annual Percent Change (APC) is significantly different from zero at the alpha = 0.05 level.

**Figure 4:**
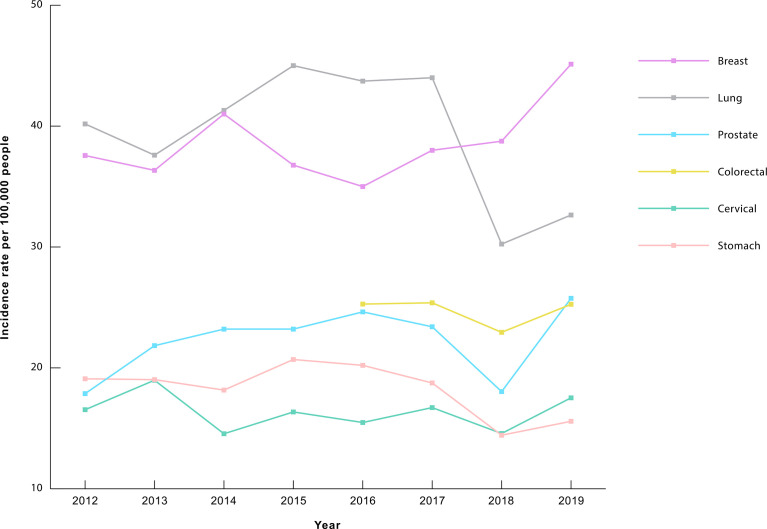
Trends in the incidence of frequently encountered cancers (2012-2019) in Armenia, rate per 100,000 people^a^. ^a^Data are adapted from the Statistical Yearbook of Armenia: Health and Healthcare, for the years 2014-2020 (3, 28, 29).

The incidence, prevalence, and mortality rates of cancer increased from 1991 until 2014, after which incidence became relatively constant, while prevalence showed an upward trend and mortality showed a slightly downward trend. A 2-joinpoint model best describes the increase in incidence rate in the past three decades. The annual percent change in incidence rate was significantly different from zero from 1997 to 2019 at a 0.05 significance level. As shown in **Figure 2**, the incidence rate has increased by 6.65% annually from 1997 to 2006, and by 2.29% annually from 2006 to 2019 (3). The drop in incidence in 2019 seen in **Figure 2** is partly due to a change in methodology as post-mortem diagnoses were excluded that year. As for the increase in prevalence rate, a 3-joinpoint model was selected to best describe the data. The annual percent change in the prevalence rate was significantly different from zero from 2000 to 2019 at a 0.05 significance level. As shown in **Figure 3**, the prevalence rate has increased by 12.04% annually from 2000 to 2003, and by 2.26% annually from 2003 to 2009 and by 6.41% from 2009 to 2019 (3). The increase in mortality rate is described by a 3-joinpoint model as shown in **Figure 2**. The annual percent change in mortality rate was significantly different from zero from 1994 to 2015 at a 0.05 significance level. Mortality has increased by 5.41% annually from 1994 to 2006 and by 2,38% annually from 2006 to 2015 (3, 23–27). The incidence rates of breast, cervical, lung, colorectal, prostate, and stomach cancers have fluctuated in the past 8 years and did not show any significant trends. **Figure 4** presents the trends in the incidence rate of these frequently encountered cancers from 2012 to 2019 (3, 28, 29).

#### Stage at Diagnosis

As of 2019, nearly half (49.6%) of all cancers are diagnosed at stages III or IV (3). Furthermore, as much as 80% of lung cancers and 70% of stomach cancers are diagnosed at these stages (3). For perspective, this can be compared to neighboring countries, such as Georgia, where around 59% of all cancers are diagnosed at a late stage (30). **Table 1** shows the percentage of cases at each stage at the time of diagnosis for common cancer types in Armenia in 2019 (3).

**Table 1:** The percentage of cases in each stage at the time of diagnosis for common cancers in Armenia, 2019.

Type of cancer	Stage I-II (%)	Stage III (%)	Stage IV (%)
Breast	76.8	6.9	16.3
Cervical	37.1	44.0	19.0
Lung	17.2	19.3	63.5
Colorectal	34.4	36.0	29.6
Stomach	30.6	26.9	42.5
Bladder	77.2	10.7	12.2
Prostate	35	28.8	36.1
All	50.4	19.5	30.1

(Data are adapted from the Statistical Yearbook of Armenia: Health and Healthcare 2020) (3).

According to GLOBOCAN 2020, Armenia was ranked third for the highest incidence of cancer in Western Asia (2). Armenia also had the highest mortality rate in the region and ranked 17^th^ highest worldwide (2). The prevalence rate was also higher in Armenia compared to other LMICs, ranking third in Western Asia (2). This is possibly due to the high prevalence of risk factors, incomplete screening strategies, as well as issues with diagnostic and treatment modalities. On the other hand, the incidence rate of cancer in Armenia is considerably lower than that of most high-income countries. This may be due to the underdiagnosis of cancer in the country and it does not necessarily suggest that the cancer burden in Armenia is lower (2).

### Risk Factors and Prevention

The cancer with the highest mortality rate in Armenia is lung cancer – a prominent risk factor of which is smoking (2, 3). About 28% of the population of Armenia smokes (51% of all males and 2% of all females) (6). Armenia’s smoking rate is higher than the world average (20%) (31). Around half of the people who smoke are predicted to die prematurely (32). While the proportion of smokers in neighboring Georgia is higher (31%), their lung cancer incidence rate is lower than that of Armenia (2, 33). Further research in the area is needed to determine whether the difference is significant. It is possible that the smoking rate among certain groups of the Armenian population, such as women or adolescents, is underestimated due to inaccurate self-reporting. Also, a large proportion of smokers in Armenia consume a higher than average amount of cigarettes per day.

Over the years, Armenia has failed to adopt proper strategies to combat tobacco use. Most notably, it failed to ensure smoke-free environments – except for schools and hospitals – and the implementation of many laws and regulations that aimed to restrict tobacco use was unsuccessful (34). That being said, Armenia was able to enforce the labeling of most tobacco products as harmful and the banning of sales to underage individuals (34).

In February 2020, the Armenian parliament approved a law that introduced further restrictions on tobacco use, including a ban on smoking in a wide range of locations (35). The law will gradually enter into force in the upcoming years, with the most notable ban to be introduced in 2022 (35). The law promises to introduce heavier sanctions upon violation compared to previous tobacco-related regulations (35).

Another common risk factor for cancer is alcohol consumption (36). High alcohol consumption increases the risk of developing cancers of the mouth, throat, larynx, esophagus, colorectum, liver, and breast (36). The last 3 cancer types have a high incidence rate in Armenia, which may be attributed to the high level of alcohol consumption in the country (3). Around 5.0% of all cancers in males and 1.8% of all cancers in females in Armenia are attributed to alcohol consumption (37). Armenia has an average rate of alcohol consumption compared to countries of the Commonwealth of Independent States (CIS), ranking behind Belarus, Russia, Moldova, Ukraine, Georgia, and Kyrgyzstan (38). Most of these countries have a higher incidence rate of alcohol-related cancers than Armenia (2), including colorectal, lip, oral cavity, throat, and esophageal cancers (2).

An unhealthy diet and physical inactivity are among the main risk factors for cancer (39). According to the 2016 national STEPS survey, about 20% of adults in Armenia are obese (14% of males and 25% of females) and 48% are overweight (6). Thus, the proportion of people with unhealthy weight is considerably higher than the world average (13% obese, 39% overweight), but closer to the European average (22% obese and 50% overweight) (40, 41). About 21% of Armenians are considered physically inactive, as they perform less than 150 minutes of moderate-intensity work per week (6). This is close to the average proportion of inactive people in LMICs, yet lower than the global average (6, 38, 42, 43). Salt intake in Armenia is twice the daily recommendation by WHO, which is 5 g daily (6, 44). The majority of Armenians (76%) eat less than the WHO recommended average of 5 servings of fruit and/or vegetables per day (6). Armenia ranks fifth among countries that consume the least amount of vegetables – followed by neighboring Georgia (45). On the other hand, Armenians consume processed meat at remarkably higher amounts than other countries in Central Asia and the South Caucasus (45). This potentially contributes to Armenia’s incidence rate of colorectal cancer, which is higher than that of any other country in said region (2).

Yet another risk factor for cancer is air pollution, which accounts for up to 30% of lung cancer cases worldwide (46, 47). Armenia ranks 23^rd^ in the world regarding air pollution and has two of the ten most air-polluted cities in Western Asia (46). The mining industry is a significant contributor to this problem (48). It is estimated that the emission of hazardous substances into the atmosphere due to mining, and other related operations, accounts for 13% of total emissions (48). The soil in towns proximal to mining sites is contaminated with several carcinogenic elements, including arsenic, lead, and cadmium (49). Moreover, about 57% of the capital city’s residents are affected by ground contamination through the use of contaminated irrigation water (50). Overall, mining operations throughout the country are poorly regulated (50).

### Screening and Early Detection

The majority of Armenia’s population is vaccinated against hepatitis B in the first year after birth (34). HPV vaccination became a part of the national immunization program in late 2017 and has since been provided to females ages 13-14 free of charge. Vaccine coverage is increasing at a slow rate and remains low at an estimated 10% (51, 52). The low uptake of this vaccine may be due to the lack of knowledge about its necessity and the spread of false information about its harm (51, 52). According to a study among parents of teenage girls, some family physicians have advised against taking the vaccine (51). Although a great challenge, this misinformation must be addressed, especially when considering that the incidence rate of cervical cancer in Armenia is the second-highest in the region (2).

Armenia first launched a systematic screening program for cervical cancer in January 2015 (53). Within one year, around 110,000 women, ages 30-60, were screened by Pap smear (around 30% coverage) (53, 54). This marked a three-fold increase compared to the number of women who underwent cervical cancer screening in 2012 (53). The 3-year coverage rate now stands at 41% among this population (55), and fails to reach a 70% coverage rate achieved by many LMICs worldwide (56, 57).

Armenia does not have a systematic breast cancer screening program. The proportion of women aged 30-60 who underwent mammography screening during the past 3 years was lower in 2012 than in 2016 (about 12%) (54). This low rate can be partially explained by the lack of free of charge mammography examination services for the general population and high-risk groups alike (54). However, in 2020, a mobile mammography screening unit was introduced to screen for breast cancer among women aged 50-70 throughout the country (58). As for the less effective ultrasound breast examination, it is offered in polyclinics free of charge but its 3-year coverage among women aged 30-60 is only 23% (54). Furthermore, the Armenia Demographics and Health Survey (DHS) of 2010 showed that 78% of women did not know how to perform a breast self-examination, despite health care providers being required to teach it to their patients during annual check-ups (59). In addition, only around 10% of women aged 30-50 reported ever receiving a manual breast examination by a health care provider (59).

Opportunistic colorectal cancer screening is also available in polyclinics (60). But because screening for colorectal cancer is recommended to be done systematically (61), a screening program will be launched for people aged 55-75 in the near future.

As for prostate cancer, only around 6% of males have had a prostate ultrasound examination at least once in the past year, which is done on an opportunistic basis, as recommended (54, 61).

With the implementation of the aforementioned screening and secondary prevention strategies, cervical tumors were detected in 15% of women who underwent screening in the last 1-3 years (54). Breast tumors were detected in 25% of women who underwent mammography examination in the last 1-3 years (54). In addition, numerous actions were taken to educate the general public about the importance of early detection of different cancer types (62). Likewise, screening guidelines were published for primary healthcare providers on providing screening for cervical cancer (63). It is worth noting that, as shown in **Figure 4**, the incidence of cervical cancer has not seen any major changes after the implementation of systematic screening in 2015. Incidence is expected to decrease through the detection and treatment of precancerous lesions, but it may be too early to see a trend yet. Nevertheless, there was a reported increase in the proportion of cases detected in early stages among females aged 30-60 during the first two years of the program (64); but such an increase did not occur in the general female population (3, 29, 54, 64–66).

A recent initiative towards the modernization of early detection strategies is the Armenian Research Infrastructure on Cancer Research (ARICE) project. ARICE is a HORIZON 2020 Twinning project between YSMU, the Medical University of Graz, Charles University Prague (CUP), and the International Agency for Research on Cancer (IARC) of WHO, which is aiming to build up a cancer biobank and a cancer management training network for establishing biomarker analysis standards for early detection of cancer specifically in the Armenian population (67).

### Diagnosis

Diagnostic methods in Armenia have undergone a major improvement during the past decade, however, challenges remain (68). Diagnostic imaging modalities are present and available to patients in Armenia at lower rates than in most Eastern European countries (69). Armenia has about 2.4 MRI scanners and at least 7 CT scanners per 1,000,000 population (69). A PET/CT scanner was recently acquired and there is a novel center for nuclear medicine within one of the medical centers in Yerevan (70, 71). Still, experts in the field argue that some of the essential cancer imaging techniques are either outdated or completely absent (i.e. the SPECT/CT scanners, most of which are old) (61). In addition, the lack of protocols substantially hinders the diagnostic process. Even though many clinicians/radiologists utilize guidelines from prominent foreign associations, no government-set protocols exist and there are discrepancies among different institutions in terms of diagnostic approaches.

As for laboratory examinations, all essential tools for laboratory diagnosis are available. Nonetheless, a major problem in this field lies in the lack of good documentation practice, as most institutions have no laboratory information management system. This leads to a greater risk of making errors and the loss of important data.

As for genetic testing for cancer, there is a specialized genetic center in Armenia, which performs analyses of the full list of somatic mutations of genes (e.g. EGFR, KRAS, NRAS, BRAF, ALK) for targeted therapies required by the international guidelines, as well as Next Generation Sequencing analyses (NGS) of the hereditary cancer gene panel (84 genes) associated with all known types of hereditary cancers, all according to the European Molecular Quality Network requirements. Nevertheless, genetic testing is conducted infrequently due to the high cost of most testing options and/or the unaffordability of possible targeted therapy.

Lastly, pathologists have highlighted many major problems in the field of histopathologic diagnostics. The main issue is the insufficient personnel and laboratory facilities required to manage large volumes of examination specimens (61). The methods many pathologists use in their practice are not consistent with internationally approved histopathologic protocols, such as proper tissue staining and slicing technique. This creates further confusion and redundancy among oncologists, as they often receive inconclusive examination results, requiring repeat biopsies (61).

### Treatment

**Figure 5:**
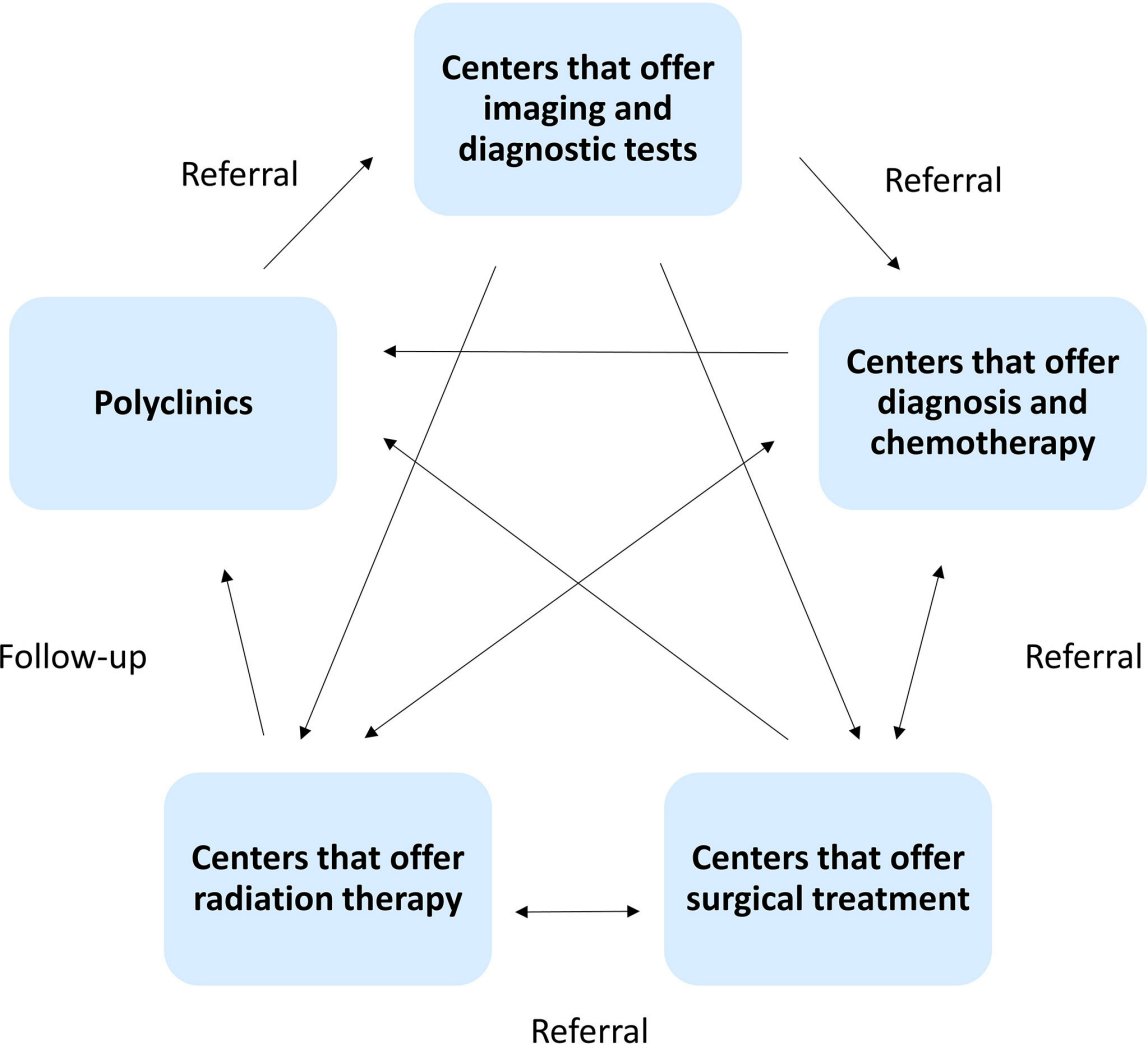
The treatment journey of a cancer patient in Armenia. This diagram aims to highlight the multiple referrals that occur in a patient’s treatment process. Not all patients go through the same process and in the same order.

All main cancer treatment modalities are available in Armenia, including surgery, radiotherapy, and medication therapy. **Figure 5** presents a simplified diagram of the process of cancer treatment in Armenia.

Surgical oncology is not a registered specialty in Armenia and many surgeons who practice the specialty are also involved in other non-oncological surgical procedures. Despite that, the National Center of Oncology has nine departments specialized in the surgical treatment of cancer (61). Most of the necessary treatment options are available and accessible, but several novel treatments, such as arterial embolization of tumors, are absent. In addition, surgeons generally do not follow a uniform national protocol, which leaves plenty of room for discrepancies.

The provision of radiation therapy is mostly centralized (61). Armenia has two linear accelerators and one Telecobalt unit, all located in the capital city, Yerevan. There used to be a Telecobalt unit in the second most populated city of Gyumri, but it was decommissioned recently in 2020. There is also one center that offers brachytherapy (61). Despite this, there are problems with the immobilization devices, which are limited and therefore often reused or used inconsistently. This increases the adverse effects of radiotherapy and directly impacts its efficiency (61).

Medical oncology in Armenia is fairly up-to-date. However, a major limitation in this field is the accessibility and registration status of medication. The national essential medicines list (EML) in Armenia includes 37 out of 62 of the antineoplastic and supportive cancer medicines listed in WHO’s EML (72, 73). However, the registration of many of the medications in the EML is not regularly renewed. Among the cancer drugs that are considered nationally essential, 30% are not registered and research shows that drugs which are not registered are mostly inaccessible to patients (74, 75). A study conducted in 2018 reported that 8 out of the 30 essential pediatric cancer medicines were not available in Armenia at the time (76). The study reported a decrease in the percentage of registered essential medicines since 2016, which highlights the lack of effort directed at solving this issue (76, 77).

The lack of proper state coverage of cancer medicines makes access to them impossible for a large majority of the population. Even when a certain part of treatment is covered by the state, clinicians report that many patients cannot pay for the non-covered part which renders their treatment incomplete. In addition to that, clinicians strongly emphasize patients’ inability to cover the cost of novel therapies. According to one clinician, only 1 out of 10 patients who need immunotherapy can cover its cost. In 2020, the state launched a program to provide the targeted therapy drug Trastuzumab free of charge for women with non-metastatic HER2/neu positive breast cancer (78). Also, pediatric cancer treatment is provided almost completely free of charge thanks to charitable organizations.

Recently, access to medicines has become even more limited due to the novel coronavirus (COVID-19) pandemic as Armenia’s drug supply routes have been suspended. This impacted the availability of some of the essential cancer medications. As a result, many treatment plans were changed by substituting the unavailable medication with available ones.

Concerning the transplantation of hematopoietic stem cells, the Hematology Center after Prof. R. Yeolyan offers autologous hematopoietic cell transplantation (79). However, allotransplantation was not available until very recently, and patients used to be redirected to foreign institutions. With the launch of allogenic transplantation in 2021, allotransplantation for cancer patients will soon be performed locally.

As discussed earlier, a lack of national guidelines poses issues on many fronts, including treatment modalities. Even those who are provided with national guidelines by the Ministry of Health rarely implement them. The ones that are used are mostly taken from the European Society for Medical Oncology (ESMO), the National Comprehensive Cancer Network (NCCN), and others. Hence, they do not correspond to the contemporaneous treatment conditions in the country. That being said, detailed guidelines for the management of 17 types of pediatric cancers were adapted and developed between 2019 and 2020, following the centralization of pediatric cancer care in the Pediatric Cancer and Blood Disorders Center of Armenia in early 2019. In addition, few centers in Armenia utilize multidisciplinary tumor boards.

Finally, several institutions in the country are involved with providing palliative care. Most of the palliative care medications included in the WHO EML are registered and available in Armenia (73). Nevertheless, disparities exist between the need and the actual availability of palliative care services (80). Palliative care in Armenia suffers from a lack of state-approved treatment guidelines, a shortage of trained personnel, a lack of awareness in patients about drug use, as well as policy and legislation-related issues (80, 81). A study conducted in 2015 revealed that while 80% of cancer patients suffered from moderate to severe pain, only 8% received a strong opioid analgesic (80). As a result of this and several other studies, the National Strategy on Palliative Care Action Plan was adopted (81). Following the approval of the action plan, oral morphine became officially registered in Armenia in mid-2018 and is now more easily accessible to patients with cancer, albeit with occasional shortages (74, 81).

**Figure 6:**
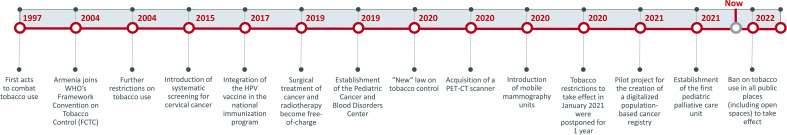
Timeline of major events in the development of cancer care in Armenia.

A timeline of major events that contributed to the development of cancer care in Armenia is shown in **Figure 6**. It is worth noting that charitable organizations have significantly contributed to the development of cancer care. In the nineties, pediatric cancer care was largely covered by the “Hilfe fur Armenia Foundation” (Germany); later on, in different periods, charities such as “Bridge of Health”, “Nvirir Kyanq”, “Ognem”, “Fund 100”, “Menq enq”, and “City of Smile” have continued to fund the treatment of patients as well as the professional development of physicians and other capacity-building projects. The City of Smile foundation is currently the largest cancer charity organization in Armenia and it has been undertaking most of the coverage for pediatric oncology in the last few years. Recently, the foundation has extended its support to also cover the diagnosis and treatment of young adults aged 19 to 25. Adult cancer patients also receive charitable support in certain cases, e.g. the Max Foundation provides Glivec^®^ free of charge for patients with chronic myeloid leukemia (CML). There are also several patient advocacy organizations and support groups, such as the Henaran Foundation, that provide legal, psychological, and social support to cancer patients in addition to financial support.

### Recommendations

Based on this review, we have designed a list of recommendations that address the major problems associated with the burden of the disease;

1. Ensure the implementation of anti-tobacco laws and regulations and encourage a healthy lifestyle. Expand the National Cancer Control Plan (NCCP) with regard to primary prevention by adding a timeline for each objective, defining every objective in measurable terms, and monitoring implementation.
2. Create a population-based national cancer registry to obtain a good understanding of the epidemiology of cancer in the country, especially with regards to culturally specific risk factors.
3. Boost early detection by increasing cancer awareness among the general population and primary healthcare providers, as well as ensuring equitable access to all screening services especially for the early detection of breast cancer.
4. Improve access to medicines by ensuring the timely registration of all those deemed essential by WHO and ensuring full cost coverage for at least those that are considered essential nationally. Regarding painkillers, improve access by raising awareness among prescribing physicians on the necessity of effective pain management among cancer patients.
5. Improve diagnosis quality by upgrading diagnostic documentation and management systems in accordance with current guidelines, as well as by ensuring the development of an appropriate workforce and facilities for histopathologic services.
6. Improve treatment outcomes by creating multidisciplinary tumor boards and participating in partnership programs with developed countries, creating national cancer management guidelines, importing novel approaches and techniques, and most importantly, monitoring the quality and adherence to guidelines.
7. Within the NCCP, define the specific role of different sectors of the government and society, such as the education sector, environmental sector, ministry of labor, NGOs, medical associations, patient advocacy groups, etc.

## Conclusion

Cancer control in Armenia has improved greatly over the past decade. Developments are observed in almost all aspects of cancer care and prevention. Still, there is plenty of room for improvement, and shortcomings are often identified by health authorities. However, rapid improvement seems somewhat impossible due to economic, cultural, and political factors.

**Note:** This article reviews the cancer situation in Armenia before the war which occurred from September 27, 2020, to November 10, 2020. The implications of the war are not taken into account in this review.

## Author Contributions

KB: conceptualization, data curation and literature search, interpretation of data, project administration, writing of the original draft, visualization (figures), validation of all data provided in the paper. TA: conceptualization, data curation and literature search, interpretation of data, project administration, writing of the original draft, visualization (figures), validation of all data provided in the paper. AMe: data curation and literature search, reviewing and editing. SS: validation of data on health care organization, interpretation of problems and suggesting of solutions, critical revision and editing of the paper. DZ: validation of data on adult medical oncology, critical revision and editing of the paper. LSaf: validation of data on adult medical oncology, critical revision and editing of the paper. LSar: validation of data on pediatric hematology and oncology, critical revision and editing of the paper. AA: validation of data on adult medical oncology, critical revision and editing of the paper. LH: validation of data on adult medical oncology, critical revision and editing of the paper. AV: validation of data on adult hematology and oncology, critical revision and editing of the paper. ATad: validation of data on risk factors and prevention, critical revision and editing of the paper. DM-N: validation of data on policy and epidemiology, critical revision and editing of the paper. PK: validation of data on cancer pathology, critical revision and editing of the paper. TSag: validation of data on radiation oncology, critical revision and editing of the paper. MK: validation of data on surgical oncology, critical revision and editing of the paper. HV: validation of data on radiology, critical revision and editing of the paper. MH: validation of data on policy and epidemiology, critical revision and editing of the paper. TSar: validation and provision of data on genetics, critical revision and editing of the paper. KS: validation and provision of data on research and genetics, critical revision and editing of the paper. DB: validation of data on genetics, critical revision and editing of the paper. ATan: validation of data on cancer care organization and management, critical revision and editing of the paper. SD: validation of data on cancer care organization and management, critical revision and editing of the paper. AMu: validation of data on health policy and regulations, critical revision and editing of the paper. GT: conceptualization, validation of data on pediatric oncology, philanthropy, and global comparisons, critical revision and editing of the paper, supervision and mentorship. SB: conceptualization, data curation, validation of all data provided in the paper, critical revision and editing of the paper, project administration, supervision and mentorship. All authors contributed to the article and approved the submitted version.

## Conflict of Interest

The authors declare that the research was conducted in the absence of any commercial or financial relationships that could be construed as a potential conflict of interest.

## Publisher’s Note

All claims expressed in this article are solely those of the authors and do not necessarily represent those of their affiliated organizations, or those of the publisher, the editors and the reviewers. Any product that may be evaluated in this article, or claim that may be made by its manufacturer, is not guaranteed or endorsed by the publisher.
