# Supplementary material for: Overview of Cancer Control in Armenia and Policy Implications
Source: Front Oncol. 2022 Jan 11;11:782581. doi: 10.3389/fonc.2021.782581 (PMC8787108; doi:10.3389/fonc.2021.782581)
Supplement: Supplementary file 1 [file Table_1.docx]

Supplementary Material

**Supplementary Table 1A: The ten most common cancers in Armenia, 2019^a^**

|  | **Type** | **Number of new cases in 2019** |
| --- | --- | --- |
| 1 | Breast | 1338 |
| 2 | Lung | 968 |
| 3 | Colorectal | 749 |
| 4 | Hematopoietic and lymphoid tissues | 547 |
| 5 | Bladder | 462 |
| 6 | Stomach | 462 |
| 7 | Prostate | 361 |
| 8 | Thyroid | 326 |
| 9 | Non-melanoma skin cancer | 288 |
| 10 | Cervix Uteri | 274 |

^a^Data adapted from the Statistical Yearbook of Armenia: Health and Healthcare 2020 (1)

**Supplementary Table 1B: The ten most prevalent cancers in Armenia, 2019^a^**

|  | **Type** | **Number of prevalent cases in 2019** |
| --- | --- | --- |
| 1 | Breast | 12261 |
| 2 | Colorectal | 4619 |
| 3 | Cervix uteri | 3327 |
| 4 | Bladder | 3193 |
| 5 | Lung | 2508 |
| 6 | Lymphomas | 2374 |
| 7 | Uterine | 2146 |
| 8 | Leukemias | 1986 |
| 9 | Stomach | 1846 |
| 10 | Non-melanoma skin cancer | 1838 |

^a^Data adapted from the Statistical Yearbook of Armenia: Health and Healthcare 2020 (1)

**Reference**

1. Andreasyan D, Bazarchyan A, Matevosyan M, Mirzoyan L, Muradyan G, Simonyan A, et al. Statistical Yearbook of Armenia: Health and Healthcare [Internet]. Yerevan, Armenia; 2020 [cited 2020 Sep 13]. Available from: https://nih.am/assets/pdf/atvk/9cebab1c6da9fe5f6dc32d31d3405803.pdf
